# Supplementary material for: UDP-Glycosyltransferases from the UGT344 Family Are Involved in Sulfoxaflor Resistance in Aphis gossypii Glover
Source: Insects. 2021 Apr 16;12(4):356. doi: 10.3390/insects12040356 (PMC8072560; doi:10.3390/insects12040356)
Supplement: Supplementary file 1 [file insects-12-00356-s001.zip › insects-1102042-supplementary.pdf]

Table S1 The RPKM values of UGT genes in the susceptible (SS) and sulfoxaflor resistance (SulR) strains of *Aphis gossypii*.

Gene name	SulR1_RPKM	SulR2_RPKM	SulR3_RPKM	SS1_RPKM	SS2_RPKM	SS3_RPKM
UGT349A2	213.004238	238.8598868	246.2497132	225.9551571	224.8156985	264.8530509
UGT344C5	123.9402349	127.3587754	118.9160569	54.78329645	57.29239383	52.38349098
UGT344D6	112.2291104	104.2823753	107.1702567	87.46596615	92.13722876	112.3021314
UGT350C3	68.35793675	63.89459276	75.62933609	63.82328113	61.84907366	70.5646948
UGT329B3	42.72416915	45.54226436	43.28778537	40.76399142	43.07038575	43.82251012
UGT344B4	88.72468826	90.41934865	87.87225483	42.88532431	36.57688473	43.79109614
UGT344F3	16.22156601	15.70025772	15.28313408	15.23452487	14.59796677	14.19552935
UGT350C2	41.50657726	42.32241888	45.34036794	46.7642334	46.24425725	56.92837343
UGT344C5	57.11679124	61.14957448	60.60182578	45.10047536	44.41305999	40.88625439
UGT350A2	29.81567973	28.33186883	25.5862786	23.02440658	24.48848673	19.66348822
UGT342C2	24.16393504	24.69723761	27.08300956	29.98241556	31.87741578	33.90253176

UGT343C3	12.6646332	12.23179961	13.31157909	13.15799723	14.20105092	10.46742261
UGT350B2	25.45965061	21.26499999	20.44245228	18.11648461	18.20050989	14.78762597
UGT344A12	24.88841455	25.31959341	25.19218306	14.83793477	13.0552365	16.48172253
UGT344M2	17.42613172	15.49320403	15.40576964	19.81874461	17.33410857	33.30774577
UGT343B2	20.76165876	17.84024846	18.47098872	14.43761467	12.28399449	15.2465554
UGT344L2	33.34248512	30.02984641	32.36921685	24.25566749	26.92023816	20.41411593
UGT342A2	10.24803043	10.65807878	9.440293194	7.792891298	7.397576792	9.042581734
UGT343A5	14.50283603	14.3300846	14.70345977	14.26313086	15.39532435	12.48621126
UGT329A5	15.47781661	16.21852723	14.88870725	15.86821364	16.39585906	13.93077436
UGT344A14	17.66002978	19.75624663	18.89780535	13.0228544	13.20041529	13.98146752
UGT344L2	21.84887516	21.69320248	24.6949257	13.42334237	10.8632478	9.960876602
UGT343A4	11.93081697	14.46411924	15.34154842	6.04788154	5.875018608	6.131972329
UGT344B4	16.77206702	15.62423783	14.36574146	7.897812771	11.14077603	11.73978585

UGT344A11	14.72542274	16.48669071	22.17047216	3.9595825	3.790958509	5.690839856
UGT330A2	7.590653354	7.411998735	7.643836438	9.316467005	7.632071853	11.2787196
UGT348A2	6.628147346	7.408592486	9.099293274	6.852478558	6.4485433	8.714419156
UGT345A2	4.039430173	4.735181787	5.043077124	4.810998987	4.188586336	9.301970795
UGT344B5	5.584588548	5.733029096	5.743002404	6.508256695	7.187597437	4.730048712
UGT344J2	2.419921295	2.197558109	2.474661105	0.872262481	0.836080752	2.495921576
